# Retinal angiotensin II and angiotensin-(1-7) response to hyperglycemia and an intervention with captopril

**DOI:** 10.1177/1470320318789323

**Published:** 2018-08-21

**Authors:** Preenie deS Senanayake, Vera L Bonilha, John W Peterson, Yoshiro Yamada, Sadashiva S Karnik, Firouz Daneshgari, K Bridget Brosnihan, Joe G Hollyfield

**Affiliations:** 1 Department of Ophthalmic Research, Cole Eye Institute, Cleveland Clinic, Cleveland, USA; 2 Department of Ophthalmology, Cleveland Clinic Lerner College of Medicine at Case Western Reserve University, Cleveland, USA; 3 Reseach Core Services (Imaging) Cleveland Clinic, Cleveland, USA; 4 Department of Urology, Kyoto Prefectural University of Medicine, Kyoto, Japan; 5 Department of Molecular Cardiology, Lerner Research Institute, Cleveland Clinic, Cleveland, USA; 6 Department of Urology (FD), Case Western Reserve University, University Hospitals Case Medical Center, Cleveland, USA; 7 Department of Surgery, Hypertension & Vascular Research, Cardiovascular Sciences Center, Wake Forest University School of Medicine, Winston-Salem, USA

**Keywords:** Angiotensin II, angiotensin-(1-7), angiotensin-converting enzyme, diabetes, blindness, retinopathy

## Abstract

**Hypothesis:**

Hyperglycemia decreases angiotensin-(1-7), the endogenous counter-regulator of angiotensin II in the retina.

**Materials and methods:**

The distribution and levels of retinal angiotensin II (Ang II) and angiotensin-(1-7) (Ang-(1-7)) were evaluated by confocal imaging and quantitative immunohistochemistry during the development of streptozotocin-induced diabetes in rats.

**Results:**

In the nondiabetic eye, Ang II was localized to the endfeet of Müller cells, extending into the cellular processes of the inner plexiform layer and inner nuclear layer; Ang-(1-7) showed a wider distribution, extending from the foot plates of the Müller cells to the photoreceptor layer. Eyes from diabetic animals showed a higher intensity and extent of Ang II staining compared with nondiabetic eyes, but lower intensity with a reduced distribution of Ang-(1-7) immunoreactivity. Treatment of the diabetic animals with the angiotensin-converting enzyme inhibitor (ACEI) captopril showed a reduced intensity of Ang II staining, whereas increased intensity and distribution were evident with Ang-(1-7) staining.

**Conclusions:**

These studies reveal that pharmacological inhibition with ACEIs may provide a specific intervention for the management of the diabetes-induced decline in retinal function, reversing the profile of the endogenous angiotensin peptides closer to the normal condition.

## Introduction

Diabetes has reached epidemic status worldwide because the incidence of diabetes increases with age and the diabetic population is living longer. Diabetic retinopathy (DR), one of the most serious microvascular complications of diabetes mellitus, produces visual impairment that often results in blindness.^1–3^ After 20 years of living with diabetes, more than 60% of patients exhibit some degree of retinopathy.^4–6^ DR is characterized by capillary dilatation and leakage, capillary occlusion, and subsequent new vessel formation. Although DR has traditionally been viewed as a disorder of the retinal vasculature, recent evidence indicates that it also affects the retina’s glial and neural cells.^7^ A major problem with DR is the lack of early subjective symptoms; by the time most patients become conscious of an abnormality in their vision, it is too late to reverse the disease process. Current treatment for DR is laser photocoagulation for neovascularization and vitrectomy for membrane proliferation and retinal detachment. In several cases, these surgical treatments have not restored vision, even though surgery appeared to be successful. New noninvasive therapeutic strategies that can prevent the development of DR or can be used from the early stages of diabetes are urgently needed. Therefore, a pharmacological regimen that could prevent the development of DR before irreversible damage occurs would be an important contribution to the management of diabetes.

Although the pathogenesis of diabetic retinal complications is not fully understood, emerging evidence implicates angiotensin II (Ang II) as an important contributor to retinal damage.^8–13^ Ang II levels are significantly higher in the vitreous of patients with proliferative DR (PDR).^14^ Ang II levels are also higher in diabetic than in nondiabetic human donor retinas.^15^ However, the levels of retinal Ang-(1-7), the endogenous counter-regulator of Ang II,^16–20^ have not been elucidated. The equilibrium between Ang II and Ang-(1-7) may be important to sustaining normal retinal function. An evaluation of both endogenous peptides is important when studying the role of the renin-angiotensin system (RAS) under normal and altered physiology and pathophysiology.

Ang II is a pro-inflammatory molecule that can contribute to diabetic complications.^21,22^ As an inflammatory mediator, Ang II enhances vascular permeability through prostaglandins and vascular endothelial growth factor (VEGF)^23,24^ and contributes to the recruitment and expression of inflammatory cells by inducing chemokines and adhesion molecules. On the other hand, Ang-(1-7) is anti-inflammatory and anti-angiogenic and thus could oppose the actions of Ang II.^19,20,25–27^

Angiotensin-converting enzyme inhibitors (ACEIs), which block the conversion of Ang I to Ang II, are widely prescribed and clinically beneficial.^28–30^ ACE inhibition salvages the visual loss caused by diabetes.^31^ ACEI treatment of diabetic rats reduced VEGF gene expression and improved vascular permeability.^32,33^ However, there is no information on the effect of ACEI treatment of diabetic rats on the profile of retinal expression of both Ang II and Ang-(1-7).

The aim of the present study was to characterize retinal Ang II and Ang-(1-7) during the development of experimental diabetes and treatment of diabetes with the ACEI captopril.

## Materials and methods

### Experimental animals

Treatment of animals conformed to the Association for Research in Vision and Ophthalmology (ARVO) Statement for the Use of Animals in Ophthalmic and Vision Research and to specific guidelines of Cleveland Clinic’s Institutional Animal Care and Use Committee. Ten-week-old female Harlan Sprague-Dawley (SD) rats (Harlan Sprague-Dawley Inc., Indianapolis, IN, USA) were housed in filter-top sterile cages that had been disinfected by cage washing. Husbandry was standard rat chow and water *ad libitum*. The light regimen in the colony room was 50-80 lux at the cage level with a 12:12 hr light–dark cycle. Experimental and control animals were housed under identical conditions.

Diabetes was induced by an intraperitoneal (IP) injection of streptozotocin (STZ; Sigma-Aldrich, St. Louis, MO, USA) 35 mg/kg in 10 mM citrate buffer, pH 4.5. Rats were weighed and their blood glucose levels determined using a OneTouch Ultra Lifescan meter (Life SCAN, Inc., Milpitas, CA). Blood glucose >250 mg/dL confirmed the diabetic phenotype. Control rats received citrate buffer (IP). Diabetes was managed with the following supportive care to prevent distress to the rats: Daily cage changes and twice-daily checks of drinking water levels; rats were examined daily by both a certified veterinary technician and a laboratory technician; blood glucose levels were determined weekly to assess the hyperglycemic status of the rats.

### Experimental protocols

#### Protocol 1

*Evaluation of the time course for retinal Ang II and Ang-(1-7) during the development of experimental diabetes in rats (n = 36 rats).* Retinal Ang II and Ang-(1-7) were evaluated at 3, 9, and 15 weeks to assess the time course of development during diabetes in Harlan SD rats. Six nondiabetic controls and six diabetic rats were studied at each time point. Rats were weighed and blood glucose levels determined at each time point prior to termination. The rats were anesthetized with urethane (1.2 g/kg, IP) and perfused *in situ* with phosphate-buffered saline (PBS) followed by 4% paraformaldehyde. Eyes were enucleated, fixed by immersion, and processed as described below for immunohistochemistry, confocal imaging, and quantitative analysis. Rats were euthanized with an overdose of pentobarbital. The eyes were further fixed by immersion with 4% paraformaldehyde-PBS. After fixation, the eyes were frozen in optimal cutting temperature (OCT) compound and stored at −80°C.

#### Protocol 2

*The effect of ACE inhibition by captopril on Ang II and Ang-(1-7) in diabetic rats (n = 15 rats).* To evaluate the effect of ACE inhibition on retinal Ang II and Ang-(1-7), comparisons were made between diabetic and nondiabetic rats after 12 weeks of treatment with captopril. Three groups of rats were included: nondiabetic rats and diabetic rats with and without captopril (Sigma-Aldrich; 42 mg/L in drinking water; *n* = 5 rats per group). Rats were anesthetized with urethane (1.2 g/kg, IP), and eyes were enucleated as described above. The eyes were further fixed by immersion with 4% paraformaldehyde-PBS, frozen in OCT compound and stored at −80°C.

### Immunohistochemistry, confocal imaging, quantitative analysis

Ang II and Ang-(1-7) were localized by immunohistochemistry and confocal imaging. Frozen sections (10 µm) were prepared from the frozen OCT eyes and stored at −80°C. Immunolocalization of Ang II and Ang-(1-7) was determined through incubation of sections with the following antibodies: rabbit polyclonal antibodies for Ang II (H-002-12, 1:200; Phoenix Pharmaceuticals, Burlingame, CA, USA) and an in-house antibody for Ang-(1-7) (CCF-Core 1, 1:200).^34^ For Ang II and Ang-(1-7) localization, sections were digested with 10% pronase (Sigma-Aldrich) in acetate buffer (calcium 5 mM and sodium 10 mM (pH 7.5)) for 15 min at 37°C and washed with PBS. Sections were blocked in PBS supplemented with 2.5% bovine serum albumin (PBS/BSA) and incubated with the primary antibodies anti-Ang II and anti-Ang-(1-7) prepared in the same solution. Antibody binding was resolved by incubation with secondary antibody coupled to Alexa Fluor 488 and Alexa Fluor 594 (Molecular Probes (ThermoFisher), Eugene, OR, USA). Secondary antibodies were prepared in PBS/BSA. Specificity of the immunostaining was verified by substituting the primary antibodies with an equivalent dilution of no immune IgGs. Slides were mounted in Vectashield containing 4’6-diamidino-2 phenylindole (DAPI) for labeling nuclei (Vector Laboratories, Inc., Burlingame, CA, USA). Sections were imaged using a Leica laser scanning confocal microscope (Leica, Heidelberg, Germany). Immunoreactivity was quantified using Image-Pro Plus 7.

### Statistical analysis

Data are presented as mean ± standard error of the mean (SEM) for continuous variables. Assuming normality of the data, analysis of variance (ANOVA) models were used to test the difference among groups over time points, with *p* value <0.05 as the level of statistical significance. For body weight, baseline weight was used as a covariate in an analysis of covariance model. If the interaction between time and group was not significant, overall differences between groups were provided. Otherwise, comparisons of groups at each time point were performed. Pairwise ad hoc comparisons and a Bonferroni correction were used to specify the differences among the three groups. SAS version 9.2 (SAS Institute, Cary, NC, USA) was used to perform all analyses.

## Results

Table 1 shows the weight gain and blood glucose levels of the rats at 3, 9, and 15 weeks after STZ treatment. At three weeks, there was no difference in the final weight between control and diabetic groups, even though blood glucose levels were significantly elevated. At later stages of diabetes (9 and 15 weeks), diabetic animals showed significantly less weight gain compared with controls. Glucose levels remained elevated during this period.

**Table 1.** Body weight and blood glucose levels of diabetic and age-matched control rats.

Experimental time point	Group	Initial weight (g)	Final weight (g)	Blood glucose (mg/dl)
3 weeks	Control	231 ± 20.5	249 ± 8.5	113 ± 25.0
	Diabetic	232 ± 9.4	252 ± 13.4	574 ± 34.6**
9 weeks	Control	242 ± 9.9	271 ± 22.8^a^,^b^	106 ± 20.6
	Diabetic	231 ± 8.5	253 ± 18.1*	564 ± 67.4**
15 weeks	Control	239 ± 8.6	282 ± 15.9^a^,^b^	120 ± 20.9
	Diabetic	228 ± 6.1	266 ± 22.3*	574 ± 40.9**

Values are expressed as mean ± standard error of the mean, *n* = 6/group. *p < 0.05,

** *p* < 0.001 vs control at the same time point.

Within group: No statistical difference was found in glucose over three time points in either nondiabetic or diabetic rats.

a Weight change over the three time points was significant only in the nondiabetic control rats (*p* = 0.008).

b In a pairwise comparison, changes in the final weight between 9 and 15 weeks in the nondiabetic controls were both greater than weight at 3 weeks (*p* <0. 001).

**Figure 1.**
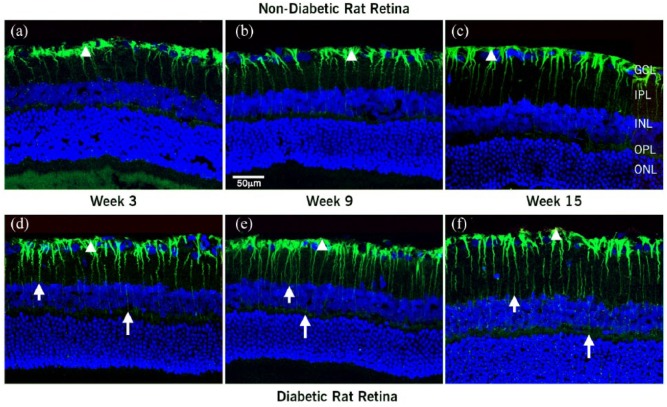
Confocal images of localization of Ang II and nuclei merge in retina sections at 3, 9, and 15 weeks after diabetes was induced: (a–c) nondiabetic and (d–f) diabetic rats. In the nondiabetic rats, Ang II antiserum labeled Müller cells. Ang II extended from the footplates of the Müller cell (arrowhead) through cell processes in the IPL into the INL. Immunoreactivity was marked in the Müller cell endfeet and extended into the cellular processes. In the diabetic retina, Ang II extended through the entire retina from the Müller cell footplates (arrowhead), cell processes in the IPL through the INL and OPL into the ONL (short and long arrow). The higher intensity and increase in extent of labeling in Müller cell processes was clear at three weeks after diabetes was induced. This pattern of labeling was maintained throughout the 15 weeks of diabetes. GCL: ganglion cell layer; IPL and OPL: inner and outer plexiform layers; INL and ONL: inner and outer nuclear layers. Scale bar = 50 µm.

**Figure 2.**
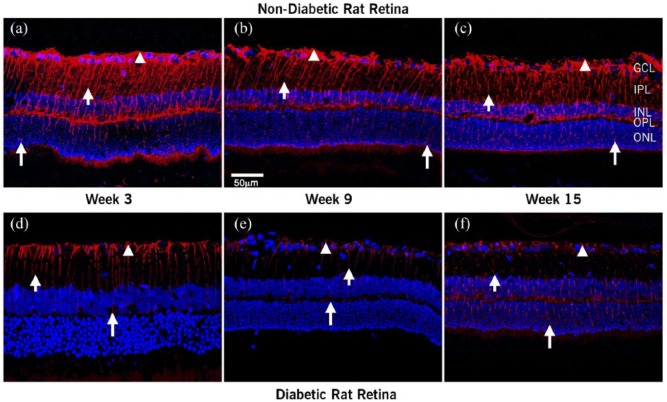
Confocal images of localization of Ang-(1-7) in retinal sections. Confocal images of Ang-(I-7) and nuclei merge in retina at 3, 9, and 15 weeks after diabetes was induced: (a–c) nondiabetic and (d–f) diabetic rats. Ang (1-7) antiserum-labeled Müller cells (arrowhead). In the nondiabetic retina, Ang-(I-7) extended from the footplates of the Müller cell (short arrow*)* through cell processes extending up to the photoreceptor layer (long arrow). In diabetic rats, the intensity and extent of labeling decreased steadily with the duration of hyperglycemia. Scale bar = 50 µm.

Some differences were observed in the distribution of Ang II and Ang-(1-7) in the different regions of the eyes analyzed. Representative images for Figures 1, 2 and 4 are shown in the manuscript. Figure 1(a) to (c) shows the time course of distribution of Ang II in the nondiabetic rat retina from 3 to 15 weeks under control conditions. Ang II immunoreactivity was localized to the endfeet of Müller cells and extended into the cellular processes in the inner plexiform layer (IPL) and inner nuclear layer (INL). In the diabetic retinas (Figure 1(d) to (f)), Ang II immunoreactivity was similarly localized to the Müller cell footplates, but extended though the entire retina, reaching the cell processes all the way into the outer plexiform layer (OPL) and into the outer nuclear layer (ONL). Diabetic rats displayed higher intensity and increased extent of Ang II labeling in the Müller cell processes compared with nondiabetic rats after three weeks of diabetes. At three weeks, Ang II staining was increased in the diabetic (Figure 1(d)) compared with nondiabetic rats (Figure 1(a)); this increased Ang II staining in diabetic rats was maintained throughout the 15-week period (Figure 1(f)).

Figure 2 shows the effects of diabetes progression on the distribution of Ang-(1-7) in the rat retina at 3, 9, and 15 weeks after the induction of diabetes (Figures 2(a)/(d), 2(b)/(e), and 2(c)/(f), respectively). In the nondiabetic retina (Figure 2(a) to (c)), Ang-(1-7) immunoreactivity extended from the footplates of the Müller cells through cell processes extending up to the photoreceptor layer. The Ang-(1-7) immunoreactivity is much more extensive than that of Ang II. In the diabetic retina (Figure 2(d) to (f)), Ang-(1-7) staining was mostly localized to the Müller cell footplates.

**Figure 3.**
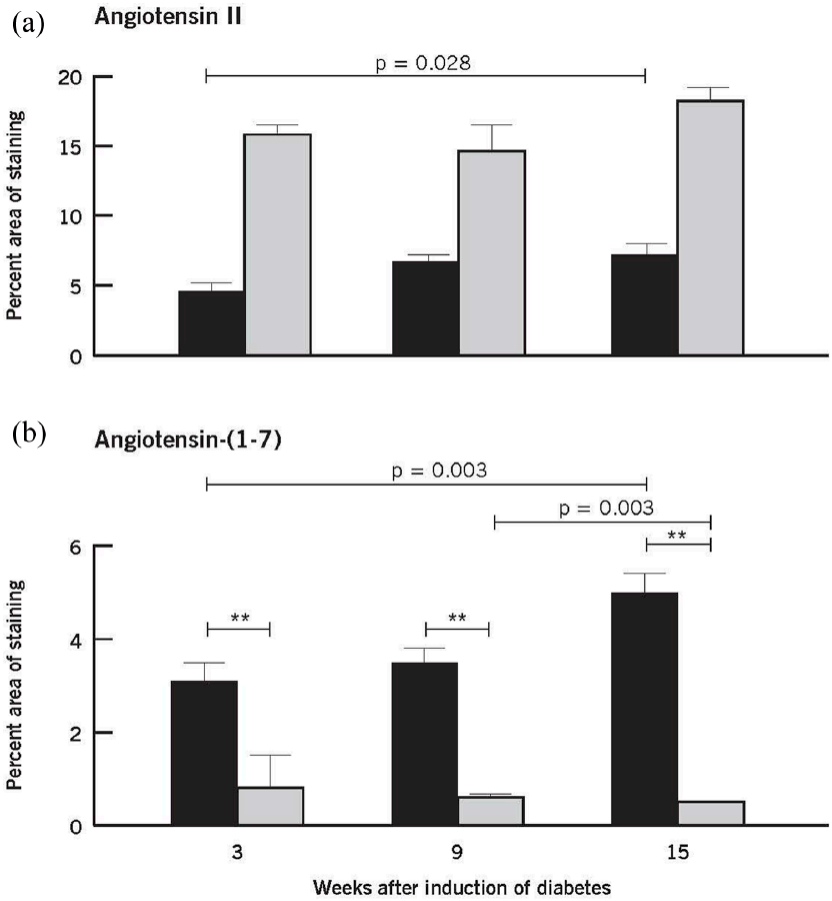
Quantitative evaluation of Ang II (a) and Ang-(1-7) (b) in nondiabetic and diabetic retinas at 3, 9, and 15 weeks after induction of diabetes. Values are mean ± SEM. Within group: Ang II and Ang (1-7) showed differences among the three time points only in the nondiabetic retinas (*p* = 0.028 and *p* = 0.003, respectively). Mean levels of Ang II in the diabetic group were statistically significantly higher than those in the nondiabetic group (**p < 0.001). Mean levels of Ang-(1-7) in the diabetic group were statistically significantly lower than those in the nondiabetic group (**p < 0.001). Nondiabetic retina (black square); diabetic retina (gray square).

Quantification of the Ang II and Ang-(1-7) staining is seen in Figure 3. The intensity and extent of Ang II immunoreactivity was markedly increased throughout all time periods as indicated by the quantification of the percent area of Ang II staining at 3, 9, and 15 weeks post induction of diabetes (Figure 3(a)). The intensity and extent of Ang-(1-7) immunoreactivity was markedly reduced throughout all time periods as indicated by the quantification of the percent area of Ang-(1-7) staining at 3, 9, and 15 weeks post induction of diabetes (Figure 3(b))

**Figure 4.**
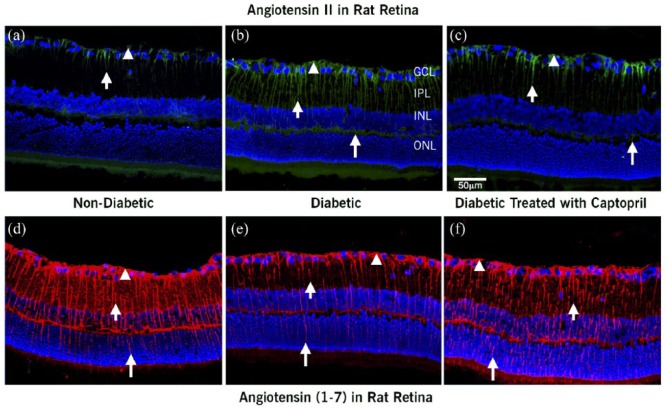
Confocal images of localization of Ang II and Ang-(I-7) and nuclei merge in retina from (a and d) control nondiabetic rats, (b and e) diabetic rats, and (c and f) diabetic rats treated with captopril. Ang II and Ang-(1-7) were localized in Müller cells (arrowhead) of nondiabetic, diabetic, and captopril-treated rats as well. Ang II was localized to the Müller cell footplates and the Müller cell processes (short and long arrows). In diabetic rats (b), the intensity of labeling was higher than in the nondiabetic controls (a); while ACEI captopril decreased the level of Ang II staining compared with diabetic, it was no different than the level of staining of Ang II (c) compared with controls. In the nondiabetic retina, Ang-(I-7) extended from the footplates of the Müller cell (arrowhead*)* through cell processes extending up to the photoreceptor layer (d, long arrow). The extent and intensity of Ang-(1-7) labeling was significantly reduced in the diabetic retina (e). Treatment of diabetic rats with ACEI restored the intensity and extent of Ang-(1-7) labeling toward levels found in control nondiabetic animals (f). Scale bar = 50 µm.

**Figure 5.**
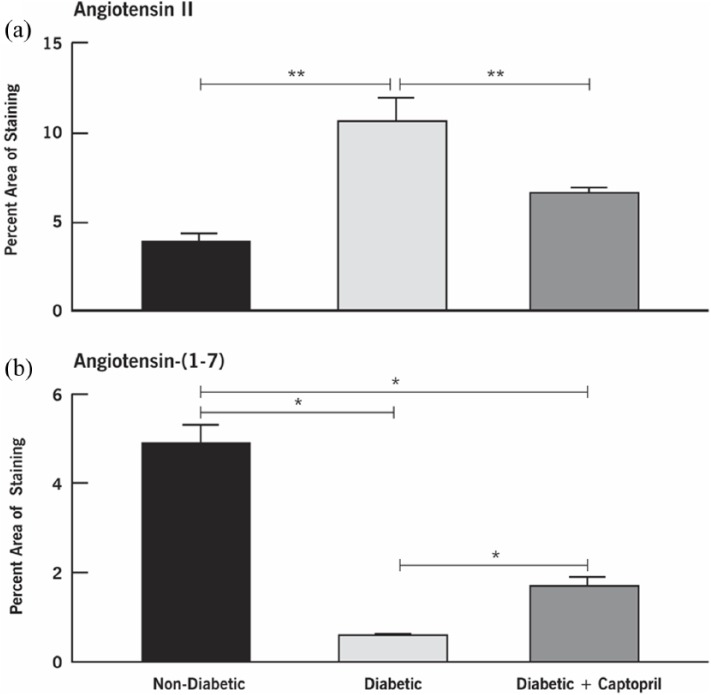
Quantitative evaluation of Ang II and Ang-(1-7) in nondiabetic, diabetic, and captopril-treated diabetic rat retinas at 12 weeks after induction of diabetes. Values are mean ± SEM. *p < 0.05, **p < 0.01 as indicated. Both outcomes showed statistical differences among the three groups.

Figure 4 shows the impact of inhibiting ACE on the distribution of retinal Ang II and Ang-(1-7) in nondiabetic and diabetic rats and in diabetic rats treated with ACEI captopril for 12 weeks (Figures 4(a)/(d), 4(b)/(e), and 4(c)/(f), respectively). The distribution of Ang II and Ang-(1-7) in the nondiabetic and diabetic animals was similar to the previously described profiles in Figures 1 and 2. In the diabetic retinas, Ang II was localized to the Müller cell footplates and processes (Figure 4(b)) and showed an increased intensity of staining compared with nondiabetic retinas (Figure 4(a)). ACE inhibition with captopril reduced the staining of Ang II in Müller cells of the diabetic retinas (Figure 4(c)). In diabetic retinas, the Ang-(1-7) staining was significantly decreased in Müller cells (Figure 4(e)) compared with nondiabetic retinas (Figure 4(d)). On the other hand, the pattern of reduced staining for Ang-(1-7) in the diabetic animals was reversed, showing increased intensity and extent of staining with ACEI treatment (Figure 4(f)). Quantitative analysis of the percent area of staining for Ang II vs. Ang-(1-7) is shown in Figure 5.

## Discussion

The present study evaluated changes in Ang II and its endogenous counter-regulator Ang-(1-7) in the retina. In nondiabetic animals, Ang II and Ang-(1-7) were both localized in Müller cell endfeet and processes, but their distribution patterns were somewhat different. Ang II was localized more toward the inner retina, whereas Ang-(1-7) spanned the entire retina from the ganglion cell layer through IPL, INL, OPL, and ONL and into the photoreceptor layer. The relatively wider distribution of Ang-(1-7) compared with Ang II was consistent with results from the immunofluorescence studies that show high levels of Ang-(1-7) receptor MAS compared with the Ang II receptor.^15,35^ Thus Ang-(1-7) may have a more important role than previously considered. In the diabetic retina, Ang II levels were increased three weeks after induction of diabetes, and the levels remained increased during the 15 weeks of diabetes. In contrast, Ang-(1-7) levels were significantly reduced three weeks after diabetes was induced and maintained at these low levels for up to 15 weeks.

The profile of the two peptides during the time course of diabetes is a novel observation of this study. The localization of Ang II and Ang-(1-7) in Müller cells is of particular interest because these cells are critically positioned between the vasculature and the neurons, and because it is suggested that they play a role in regulating the retinal molecular environment, which may be compromised early in the course of DR. Under continued hyperglycemic stress and continued declining levels of Ang-(1-7), the activity of increased Ang II will proceed unabated.

The localization of increased Ang II in Müller cells is an important finding in view of the potential role of Ang II in the initial phase of the pathogenesis of DR. In reactive Müller cells, angiotensinogen, an acute-phase protein, is upregulated in experimental diabetes.^36^ This upregulation could contribute to elevated Ang II levels and is important in view of the potential role of Ang II in the pathogenesis of DR.^37,38^ Ang II levels are significantly higher in the vitreous of patients with PDR.^14^ Ang II levels are also higher in diabetic than nondiabetic human donor retinas.^15^ In high-glucose microenvironments, Ang II increased in retinal Müller cells by 10-fold.^39^ As an inflammatory mediator, Ang II enhances vascular permeability through prostaglandins and VEGF^23^ and contributes to the recruitment of inflammatory cells by inducing chemokines and adhesion molecules. Our data suggest that Ang II may be an important factor in the activation of VEGF; this possibility has important implications since VEGF is the current anti-DR therapeutic target.

Ang-(1-7) has properties antagonistic to Ang II and is considered to be the endogenous counter-regulator of Ang II.^16–20^ Ang-(1-7) and its receptor MAS are both localized in retinal Müller cells.^15,35^ Intraocular administration of adeno-associated virus- mediated gene delivery of Ang-(1-7) to diabetic rats and mice attenuated diabetes-induced retinal vascular leakage, presence of infiltrating inflammatory cells, and oxidative damage to the retina.^25^ In rabbits, intravitreal treatment with Ang-(1-7) decreases intraocular pressure without affecting aqueous humor outflow.^40^ Although there are few data on the function of Ang-(1-7) in the retina, studies have shown a role for Ang-(1-7) in diabetes that involves other organs. An emerging protective role of Ang-(1-7) in experimental diabetic nephropathy has been reported. In diabetic hypertensive rats, Ang-(1-7) treatment exerted a renoprotective effect that correlated with the reduction in NADPH oxidase activity.^41^ In type 2 diabetic KK-A^y^/Ta mice, Ang-(1-7) attenuates Ang II-stimulated NADPH oxidase-mediated glomerular injury.^42^ Chronic administration of Ang-(1-7) improves proteinuria and ameliorates structural alterations (fibrosis and nephrin loss) in the kidney of spontaneously hypertensive stroke-prone rats, independently of local Ang II modifications.^43^ In addition, chronic treatment of Zucker diabetic fatty rats with Ang-(1-7) is associated with a reduction of systolic blood pressure, oxidative stress, and inflammatory markers.^44^ In addition, the anti-angiogenic properties of Ang-(1-7) have been shown to effectively regulate the actions of VEGF, the prime pathological entity and target for drugs to treatment DR and cancer.^45–47^ For example, mice with prostate metastases that were infused with Ang-(1-7) showed levels of VEGF below the detection limit compared with controls, which showed significantly increased circulating levels of VEGF. In vitro studies by Krishnan et al.^47^ have also shown that the Ang-(1-7) receptor antagonist D-Ala7-Ang-(1-7) could block the secretion of VEGF by transfected PC3 cells. The above studies support the concept of Ang-(1-7) as a novel target for the treatment of diabetes-induced changes.

Captopril is a sulfhydryl ACE inhibitor initially used in the treatment of hypertension and heart failure. However, later studies showed that it was protective against retinopathy and nephropathy.^48^ Specifically, captopril has been reported to stimulate retinal Na,K-ATPase activity in a diabetes model,^49^ to inhibit the diabetes-induced accumulation of glucose,^50^ to block retinal capillary degeneration and inflammation in the early stages of DR,^51^ to inhibit retinal neovascularization in oxygen-induced retinopathy mice,^52,53^ to block the stimulation of the angiotensin II type 1 receptor (AT1-R), and to attenuate the subsequent ischemic retinal damage in a rat model of retinal ischemia.^54^ In the present study, we investigated the effects of captopril on its endogenous peptide Ang II. We determined the distribution of retinal Ang II and its endogenous counter-regulator Ang-(1-7).

Captopril treatment was able to reduce the expression and levels of Ang II, while at the same time increasing the expression and levels of Ang-(1-7). ACEIs, which block the conversion of Ang I to Ang II, are widely prescribed and clinically beneficial. ACE inhibition salvages the visual loss caused by diabetes.^31^ ACE inhibitor treatment of diabetic rats reduced VEGF gene expression and improved vascular permeability.^32,55^ Under ACE inhibition, it is anticipated that Ang I will accumulate and Ang II will be reduced. Thus, in our study, the reduction in Ang II with ACEI was expected. However, it should be pointed out that levels of Ang II persisted after ACEI, a finding that is consistent with the possibility that other pathways could be contributing to the synthesis of Ang II. Similar findings have previously been described ^56^ and could be due to alternate synthetic pathways for the synthesis of Ang II, such as the enzymatic activity of chymase, cathepsin, or tonin.^57–59^ ACE, chymase, and cathepsins are present in the eye.^60–63^ ACE and chymase contribute to the formation of Ang II in the vitreous of patients with vitreoretinal disease.^64^ These data suggest that the beneficial actions of ACE inhibitors may not be a simple direct effect of Ang II inhibition, but may also consist of an increase in Ang-(1-7). ACE inhibition upregulates ACE2, a homolog of ACE^65^ that facilitates the synthesis of Ang-(1-7). ACE2 is present in both the rodent^66^ and human retina.^12^ In addition, ACE contributes to the breakdown of Ang-(1-7).^67^ Thus, ACE inhibition would result in an increase of Ang-(1-7) as well.

The prime anti-DR pharmacological target is VEGF, a major pathological factor in the promotion of DR. VEGF exerts potent effects on endothelial cell growth and vasopermeability.^32,68^ Ang II is a potent stimulant of VEGF.^69–71^ Maintaining the optimal physiological threshold for Ang II may be a more efficient and direct way to prevent VEGF-related pathologies in the retina. New noninvasive therapeutic strategies that can prevent the development of DR or be used from the early stages of diabetes are urgently needed. Therefore, our strategy of focusing on enhancing the endogenous Ang-(1-7) activity toward normal levels before irreversible damage occurs could prevent the development of DR triggered by overreactive Ang II. Our data suggest that enhancing the endogenous Ang-(1-7) concentration for the management of diabetic retinal pathology using the Ang-(1-7) agonist may be an improved therapeutic strategy to increase selectivity and specificity of targeting the RAS to prevent progression of retinal diabetic pathology such as early neural cell apoptosis and upregulation of VEGF leading to visual loss. This would be an important contribution to the therapeutic management of retinal diabetic diseases.

Although DR has traditionally been viewed as a disorder of the retinal vasculature, recent evidence indicates that DR also affects the glial and neural cells of the retina.^7^ The principal glial cell of the retina is the Müller cell, a specialized radial glial cell spanning the entire depth of the retina. Through its spatial arrangement, the Müller cell intercalates between the vasculature and the neurons. Müller cells play an important role in the uptake of glucose from the circulation, its metabolism, and transfer of energy to neurons. These crucial functions are interdependent.^72,73^ In view of their intricate metabolic interdependence, dysregulation of a number of cell functions in Müller cells can be anticipated under hyperglycemic conditions. Local vascular leakage and hyperplasia of the Müller cells are some of the earliest structural changes observed, long before overexpression of glial fibrillary acidic protein occurs in diabetic retina.^74^ The importance of understanding the tissue- and cell-specific biosynthesis of Ang II is highlighted by the differential sensitivity of bovine retinal endothelial cells (58% inhibition of intracellular accumulation of glucose) and retinal Müller cells (0% inhibition of intracellular accumulation of glucose) to the ACEI captopril under hyperglycemic stress.^50^ Most reports of anti-Ang II intervention in diabetes have focused only on the actions of pharmacological agents, while the basic underlying mechanisms of the RAS, the therapeutic target that is involved in the pathogenesis of retinal disease, has not been addressed.

## Conclusions

The current study reveals that retinal Ang II is significantly increased and Ang-(1-7) is significantly reduced in experimental diabetes. ACEI treatment restores Ang-(1-7) levels toward normality and reduces Ang II. Pharmacological inhibition, coupled with the studies in experimental diabetes, suggests that (1) there is a shift in the profile of angiotensin peptides in diabetes, with Ang II the primary peptide, and (2) with treatment, there is a reversal, with Ang-(1-7) becoming the predominant peptide. Thus, enhancing endogenous Ang-(1-7) may be a more specific intervention for the management of the diabetes-induced decline in retinal function.
